# A treatment study of canine symmetrical onychomadesis (symmetrical lupoid onychodystrophy) comparing fish oil and cyclosporine supplementation in addition to a diet rich in omega-3 fatty acids

**DOI:** 10.1186/s13028-014-0066-y

**Published:** 2014-10-04

**Authors:** Martine L Ziener, Ane Nødtvedt

**Affiliations:** Fredrikstad Animal Hospital, Wilbergjordet 2, Fredrikstad, NO-1605 Norway; Department of Production Animal Clinical Sciences, Norwegian University of Life Sciences, PO Box 8146 Dep, Oslo, NO-0033 Norway

**Keywords:** Randomized treatment trial, Symmetrical onychomadesis, Symmetrical lupoid onycdystrophy (SLO), Cyclosporine, Fish oil

## Abstract

**Background:**

Treatment of symmetrical onychomadesis (symmetrical lupoid onychodystrophy) is a challenging task for dermatologists. The acute phase is characterized by sloughing of claw plates and loose claws have to be removed and secondary infections treated. The goal of long-term treatment is to allow claws to re-grow with normal quality and to achieve life-long lack of recurrence. The aim of this randomized treatment trial was to see if adding fish oil or cyclosporine to a diet rich in omega-3 could improve the treatment outcome of symmetrical onychomadesis in Gordon and English setters. All dogs were fed Eukanuba Veterinary Diets Dermatosis® exclusively during the six month treatment trial. The treatment outcome was measured as the change in number of healthy claws during treatment, as well as the long-term effect on hunting ability and recurrence of onychomadesis. The hypothesis was that cyclosporine provides a stronger and different immune modulating property than fish oil and therefore would give a better treatment outcome in dogs with symmetrical onychomadesis eating a diet rich in omega-3 fatty acids.

**Results:**

Six Gordon setters and one English setter were treated with 5 mg/kg cyclosporine once daily for six months and seven Gordon setters were treated with 10 ml Dr Baddaky fish oil® once daily for six months. All dogs were evaluated every month and the numbers of healthy claws were recorded.

There was a statistically significant improvement in the number of healthy claws after six months of treatment with a median increase of 13.5 claws for both groups. However, there was no statistically significant difference between the two treatment groups regarding the improvement in number of healthy claws, as assessed using the Wilcoxon rank-sum test (*P* = 0.15). Dogs in the cyclosporine group had a median increase of 10 healthy claws after six months of treatment while the median for the fish oil group was 14. Long-term cure was not achieved with either treatment.

**Conclusion:**

Cyclosporine and fish oil appeared to be equally effective in treating symmetrical onychomadesis when the dog is fed a diet high in omega-3.

## Background

Dogs often damage their claws during hunting, but with symmetrical onychomadesis all claws slough off within three months and when the claws grow back, they are brittle and misshapen (onychodystrophy). The dogs experience pain in the acute phase of the disease. Symmetrical onychomadesis also diminishes the dogs’ hunting abilities and welfare through the lack of functional claws [1]. Previously, symmetrical onychomadesis was named canine symmetrical lupoid onychodystrophy (SLO) and was first described by Scott *et al.* [2]. The disease was reported to affect different breeds of dogs and mainly dogs between three and eight years of age. According to Scott *et al.* [2], affected dogs shed their claws (onychomadesis) without any signs of systemic disease and the claws are dystrophic when they grow back. Histopathological examinations of claw biopsies showed band-like subepidermal infiltrates predominantly along the dorsal aspect of the claw [2]. The disease is thought to be quite rare in most breeds, but Ziener *et al.* [1] described a high prevalence among Gordon and English setters in Norway.

Symmetrical onychomadesis is a difficult disease to treat for a veterinary dermatologist. The re-growth of claws and improvement takes time and is hard to monitor accurately. Removal of damaged claw plates during anaesthesia and treatment of secondary bacterial infections are essential in the acute phase of the disease. The goal of the long-term treatment is to encourage re-growth of normal quality claws and to prevent recurrence of onychomadesis. From a dermatological point of view, a dog is cured of the disease if it develops new claws of normal quality with no recurrence of onychomadesis during that dog’s lifetime. However, a significant proportion of owners will report the dog as “cured” as long as the dog does not have painful relapses of onychomadesis, even though it still has onychodystrophy.

Traditionally, different immune-modulating drugs have been used to treat dogs diagnosed with symmetrical onychomadesis and complete or partial remission has been achieved. Bergvall [3] demonstrated positive effects of omega-3 and omega-6 supplementation and other treatment plans have included the use of tetracycline/niacinamide, prednisolone, pentoxifylline and azathioprine [1-5]. Amputation of claws has also been used to treat symmetrical onycomadesis with good results, but this might be considered an overly aggressive treatment option [6]. In addition, a pilot study using cyclosporine in three dogs was conducted prior to initiating the current treatment trial. All dogs had re-growth of normal claws after treatment with cyclosporine for six months (unpublished data).

The aim of this randomized treatment trial was to compare fish oil supplementation and cyclosporine for the treatment of symmetrical onychomadesis in Gordon and English setters fed a diet rich in omega-3. The effect of treatment was measured by comparing the number of healthy claws in each dog before and after six months of treatment and a follow-up survey was done four years after the first dog was included in the study. The hypothesis was that dogs fed a diet rich in omega-3 would have a better treatment outcome by adding cyclosporine than fish oil, given the more pronounced and different immune modulating properties of cyclosporine compared to fish oil. To our knowledge this is the first randomized treatment trial comparing different treatment regimens in symmetrical onychomadesis.

## Materials and methods

### Study population and design

Twelve Gordon setters and one English setter diagnosed with symmetrical onychomadesis were recruited for a treatment trial at the first author’s clinic in Norway from 2008 until 2010. Participation was based on informed owner consent. The execution of the project was in compliance with ethical regulations at the Norwegian University of Life Sciences. All dogs were fed Eukanuba Veterinary Diets (EVD) Dermatosis® exclusively from the time of initiation of treatment and for the following six months. In addition, the owners were allowed to give their dogs’ potatoes and low fat white fish, as these were ingredients in the diet, but nothing else. EVD Dermatosis® was fed as an elimination diet during the study because neither of the owners had previously given their dog catfish or herring, which are the protein sources in EVD Dermatosis®.

Allocation to treatment groups was based on a systematic random procedure. This means that the first dog was allocated to treatment group based on the toss of a coin and subsequently every other dog entering the study received either fish oil (Dr Baddaky fish oil®, Dr. Baddaky AS, Skotterud, Norway) or cyclosporine (Atopica®, Novartis, Oslo, Norway) to produce groups of equal size. In both groups, the treatment was administered per os by the owners. For practical reasons, the treatment allocation was not blinded to the owner or to the responsible veterinarian.

A thorough clinical and dermatological examination of all dogs included in the study was performed. A medical history was obtained with special emphasis on ruling out previous skin diseases as a possible cause for the symmetrical onycomadesis. The number of claws without visible signs of disease was recorded.

All dogs were placed under short time general anaesthesia to remove affected claw plates and inspect claw beds at initial presentation. Samples for bacterial culture and antimicrobial sensitivity were obtained from the dogs that were not on antibiotics when included in the study. Bacterial culture and sensitivity testing are recommended as a part of the diagnostic workup for dogs with symmetrical onychomadesis [7]. All dogs had routine serum biochemistry and complete blood counts performed. A thyroid gland profile including thyroid stimulating hormone (TSH), total thyroxin (TT4), free thyroxin (FT4) and cholesterol was also obtained from all dogs.

The dogs received carprofen 2 mg/kg BID for five days and amoxicillin clavulanic acid 10 mg/kg BID for fourteen days following the removal of claw plates. Additionally, the owners were instructed to use socks on the dog’s paws as long as the claw beds were sore and to wash the paws with antibacterial shampoo daily if exudate was present.

**Figure 1:**
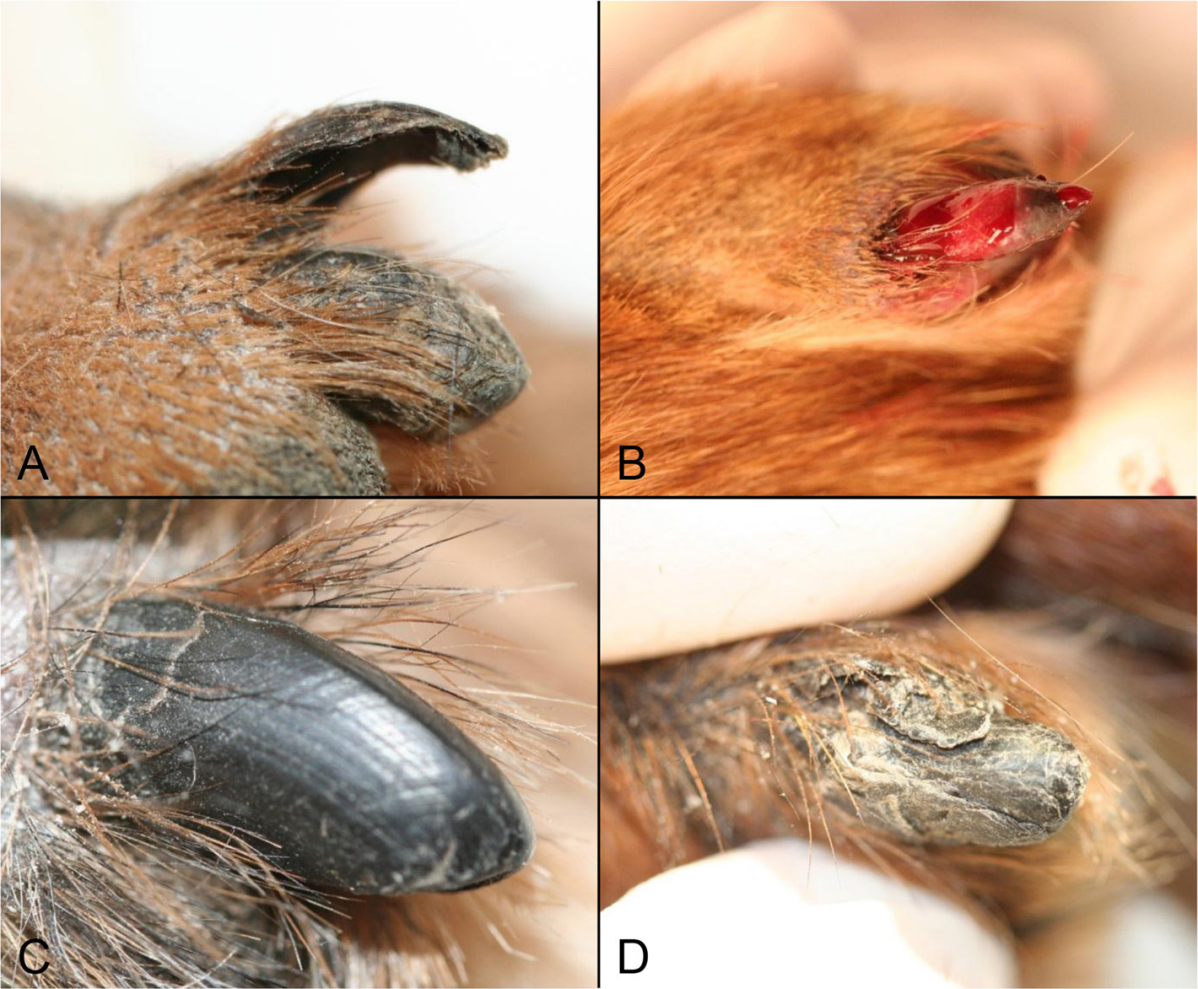
**Onychomadesis in Gordon setter. A)** Claw before treatment (onychomadesis); **B)** Claw bed after removal of horn layer; **C)** Normal claw after treatment; **D)** Onychodystrophy after treatment. Figures **A** and **B**: dog no. 3 in the cyclosporine group; Figure **C**: dog no. 5 in cyclosporine group; Figure **D**: dog no.7 in the fish oil group.

The diagnosis was based on clinical signs of symmetrical onychomadesis on several claws (Figure 1A and B) and by exclusion of other dermatological diseases as the cause of onychomadesis. Only English and Gordon setters with symmetrical onychomadesis on several claws were included in the study. All dogs which had previously received any form of immune suppressive therapy and all dogs showing only single digit onychomadesis where excluded from this study.

### Follow up

All dogs included in this study were presented for clinical evaluation once monthly for six months. During these examinations, all claw beds were inspected and the absence or presence of re-growth of claw plates was recorded. Photo documentation of re-grown claws was performed. Each claw was either characterized as normal (Figure 1C) or as having signs of onychodystrophy (Figure 1D). For every visit the number of claws that had become normal was recorded. The outcome of the treatment trial was the difference between the number of normal claws for each individual at presentation and after six months of treatment. After six months the medication was discontinued and feeding of the dog’s original diet resumed. In 2012, four years after the first dog was included in the study, a follow-up questionnaire was mailed to the owners. The owners were asked to describe their dog’s claws as normal, misshapen (dystrophic) or with recurrence of onychomadesis. The dog’s hunting abilities after onychomadesis was recorded. Furthermore, if the dog was not alive, the reason for death/euthanasia was recorded.

### Statistical methods

The change in number of healthy claws at presentation and after six months of treatment was compared using the Wilcoxon rank-sum equality of populations test. Furthermore, the effect of treatment in the two groups was compared and the same test was also used to evaluate if the sex of the dog influenced the treatment outcome. The software package Stata/SE version 11 (Statacorp, College Station, Texas) was used for the analysis. The cut-off for statistical significance was set to *P* < 0.05. A post-hoc power calculation was performed using JavaStat (http://statpages.org/postpowr.html).

## Results

Twelve Gordon and one English setter diagnosed with symmetrical onychomadesis were included in the treatment trial. The dogs were aged between three and seven years when entering the study and this was their first appearance of onychomadesis. The sex, age and weight distributions, as well as the long-term outcome for dogs in the two treatment groups are presented in Tables 1 and 2.

**Table 1:** **Demographic data from seven dogs diagnosed with onychomadesis allocated to cyclosporine treatment**

**Dog**	**Sex**	**Age of onset**	**Weight**	**Proportion of normal nails when entering study**	**Proportion of normal nails after six months of treatment**	**Long term outcome; Claw outcome; 1 = Still episodes of onychomadesis 2 = Onychodystrophy with out onychomadesis 3 = Normal claws**	**Time of follow up in years**	**Long term outcome: Is the dog used as a hunting dog?**
1	Male	3	23	6/18	18/18	Euthanized after six months	4	
2	Female	6	20	0/18	18/18	2	4	No
3	Female	4	18	5/18	15/18	1	3	Yes
4^#^	Female	6	24	10/16	7/16	2	4	No
5	Female	3	19	0/18	6/18	3	3	Yes
6*	Female	5	19	0/18	15/18	2	3	Yes
7	Male	7	23	10/18	17/18	2	3	Yes
Average		5	21	5/18 (median)	15/18 (median)	2 (median)	3.4 (3median)	

^#^Dog number four had two digits amputated.

*Dog number six was an English setter.

**Table 2:** **Demographic data from seven Gordon setters diagnosed with onychomadesis allocated to fish oil treatment**

**Dog**	**Sex**	**Age of onset**	**Weight**	**Proportion of normal nails when entering study**	**Proportion of normal nails after six months of treatment**	**Long term outcome; Claw outcome; 1 = Still episodes of onychomadesis 2 = Onychodystrophy without onychomadesis**	**Time of follow up in years**	**Long term outcome: Is the dog used as a hunting dog?**
1	Female	6	21	0/18	14/18	1	4	Yes
2	Male	6	22	0/18	16/18	1	4	Yes
3	Female	4	17	0/18	13/18	1	4	Yes
4	Male	7	19	4/18	18/16	1	4	Yes
5	Male	3	20	0/18	12/18	1	3	Yes
6	Female	7	15	0/18	14/18	2	3	No
7	Female	7	19	10/18	14/18	2	2	No
Average		6	19	0/18 (median)	14/18 (median)	1 (median)	3.4 (median4)	

Dog no. 3 in the cyclosporine group is the same dog as dog no. 7 in the fish oil group. This dog had normal claws for six months after finishing treatment with cyclosporine, before it relapsed with symmetrical onychomadesis on all claws. The dog then entered the fish oil supplementation group following random allocation.

A skin biopsy was performed in dog no. 6 from the fish oil group because of mild alopecia when included in the study. The histopathology report showed mild atrophic dermatopathy and mild superficial perivascular dermatitis. In general, the dogs showed only minor changes in albumin, globulin, alanine transferase (ALT), alkaline phosphatase (ALKP) and glucose. These were considered clinically irrelevant.

Dog no. 7 in the cyclosporine group was diagnosed with hypothyroidism when it was included in the study, based on low serum TT4, FT4, high TSH and cholesterol values. In this dog, a euthyroid state was maintained adequately by supplementation of levothyroxine (Levaxin®, Nycomed Pharma, Oslo, Norway). Dog no. 4 in the cyclosporine group had values of TT4 of 52 nmmol/l (16–46 nmmol/l) and tested thyroglobulin autoantibodies (TgAA) negative. Dog no. 6 in the fish oil group had TT4 of 47 nmmol/l (16–46) and she was not tested for TgAA.

All dogs had paronychia associated with onychomadesis. Bacterial culture and antimicrobial sensitivity analysis was obtained in nine dogs; the remaining dogs were already on antibacterial treatment upon entering the study. The results showed three samples with beta toxin producing *Staphylococcus* sp. and one with *S. pseudintermedius* from the dogs in the cyclosporine group. From dogs in the fish oil group, there were two samples with a mixed culture of Gram positive and Gram negative bacteria and one sample with *Streptococcus canis*.

### Adverse effects of cyclosporine and fish oil

None of the owners of dogs in the fish oil group reported adverse effects of the treatment. In the cyclosporine group, three owners reported that their dogs were vomiting during the initial week of treatment, but this resolved within the following four to five days, during which a small amount of food was given with the drug.

### Outcome

All dogs, except one in the cyclosporine group, showed an improvement in the number of normal claws during the course of the study period. The median number of normal claws when entering the study was 5/18 for the cyclosporine group and 0/18 for the fish oil group. After six months, the median numbers of normal claws where 15/18 in cyclosporine group and 14/18 in fish oil group (Tables 1 and 2). There was a statistically significant improvement in the number of healthy claws after six months of treatment with a median increase of 13.5 claws for both groups. However, there was no statistically significant difference between the two groups regarding the differences in number of healthy claws after six months of treatment as assessed using the Wilcoxon rank-sum test (*P* = 0.15). Furthermore, the change in number of healthy claws over time did not differ by sex (*P* = 0.89). A post-hoc power calculation showed that the power of this study to detect a difference in number of healthy claws of four between the fish-oil and cyclosporine groups was 30%. By doubling the sample size the observed difference could be detected with a power of 80% and a *P* of 0.05.

None of the owners reported any recurrence of symmetrical onychomadesis in the first month after their dog discontinued treatment and where fed their original diet.

Four years after the first dog was included in the study a questionnaire was mailed to the owners. In the cyclosporine group; one dog had been euthanized due to onychomadesis and aggressive behavior, one dog had normal claws, one still had recurrence of onychomadesis and four dogs had onychodystrophy. Four of the seven dogs were used for hunting (Table 1).

In the fish oil group; two dogs had onychodystrophy and five dogs had recurrence of onychomadesis. Five of the seven dogs were used for hunting (Table 2).

None of the dogs, in either group, had received any further treatment for their claw disease after completing the trial.

## Discussion

The primary aim of this randomized treatment trial was to evaluate if adding cyclosporine to a diet rich in omega-3 could improve treatment outcome regarding canine symmetrical onychomadesis compared to adding fish oil to the same diet. A second aim was to observe the course of the disease on a long term basis after withdrawal of treatment.

Evaluating the outcome in a prospective treatment study of symmetrical onychomadesis is challenging, because within the same dog, claws will show different stages of disease at any given time. If the inclusion criteria of this treatment trial had been absence of normal claws, the groups would have been more comparable at the start and hence the results easier to interpret.

Therefore, in order to minimize the possible effect of any difference between groups at inclusion into this study, the change in number of normal claws was used to assess treatment success. No difference in the change of number of normal claws was detected between the two groups. This could be due to low effect of adding cyclosporine to a diet high in omega-3 when treating symmetrical onychomadesis.

It was considered necessary to have a treatment period of six months for studying a disease with such a long recovery phase. The results could potentially have been different if the treatment period had been longer or shorter than six months, but earlier studies have reported efficacy of treatment within a time period of three to four months [4].

To assess the natural course of the disease without treatment, a follow up was done four years after the first dog was included in the study. Owners reported that even though none of the dogs were on any treatment, it seemed that the dogs with recurrence of onychomadesis or onychodystrophy showed less sign of pain and lameness compared to what was seen during the initial acute phase of the disease. In previous studies, effect of treatment has been reported as excellent, good, partial or poor, based on owner interviews [4]. Many owners and their dogs will consider a good response to treatment to be absence of painful relapses of onychomadesis, despite the claws showing signs of onychodystrophy. In medical terms a dog with symmetrical onychomadesis should be classified as “cured” if it has no recurrence of sloughing of claws for the remainder of its life, as well as complete re-growth of normal claws. In this study only one dog in the cyclosporine group was still “cured” after four years. The number of “cured” dogs could probably have been higher if the dogs were kept on life- long treatment, but the question is if the dogs need this, if they only have onychodystrophy without pain and lameness. Perhaps a diet change to a diet rich in omega-3 might be enough as a long term treatment in the majority of cases, as long as the affected claws are removed in the acute phase of the disease. The results could also have been different in heavier breeds known to be predisposed to symmetrical onychomadesis such as Rhodesian ridgeback and giant schnauzers. These breeds might possibly need life-long treatment because they have a higher body weight than setters and therefore could show more pain upon recurrence.

Because Mueller *et al.* [5,7] showed that symmetrical onychomadesis could be due to food allergy in two cases, all the dogs in this trial where fed the same diet. To rule out food responsive symmetrical onychomadesis all the dogs were fed their original diet after completion of the trial, and none of the owners reported any recurrence of symmetrical onychomadesis within the first month. It was also important to assure that all dogs participating in the study got the same amount of eicosapentaenoic acid (EPA) and docosapentaenoic acid (DHA) from the diet. A dose of EPA/DHA at 50–250 mg/kg body weight appears to have an anti-inflammatory effect [8]. The mean weight of the dogs participating in the study was 20 kg and a total dose of 1–5 g EPA/DHA daily would therefore be sufficient to anticipate a treatment effect. In this study each dog had a daily intake of 3.3 g EPA/DHA from the diet. Every dog ate an average of 300 g food and in EVD Dermatosis® the EPA level is 0.9% per gram and the DPA level is 0.2% per gram.

In the fish oil group each dog had a daily intake of 2.7 g EPA/DHA from the fish oil supplementation. Dr Baddaky fish oil® contains 165 mg EPA/ml, 106 mg DHA/ml and 1.0 mg vitamin E/ml and each dog in the fish oil group received 10 ml daily for six months.

Omega-3 fatty acids, in the form of fish oil, have previously been shown to have a good effect against symmetrical onychomadesis and are relatively inexpensive with few side effects [5]. It is reported that neutrophils and macrophages obtain a higher concentration of EPA and DHA in cell membranes when dogs are fed fish oil. This might contribute to modulating the immune response so that less potent inflammatory mediators are produced. Furthermore, the expression of MHC class I and II on cell surfaces decreases in animals fed a diet rich in omega-3 fatty acids [8-11].

Cyclosporine was chosen as a therapeutic agent for comparison in this study because it had shown good response in the pilot study and has previously been used to treat canine immune mediated diseases such as atopic dermatitis and anal furunculosis [12,13]. Cyclosporine is a potent immune suppressive agent that has been shown to inhibit the activation of most cells involved in cell-mediated immunity. The compound does not have major impact on humoral immune response in the short term.

Two dogs in the cyclosporine group and one dog in the fish oil group had complete re-growth of normal claws after six months of treatment. Twelve of the thirteen dogs had a higher number of normal claws at the end of the treatment period. To which extent the omega-3 from the EVD® diet contributed to the outcome of this treatment trial is unknown, because a negative control group that did not got the diet was not included in the study design.

Potential selection bias of this study was avoided through the random allocation of dogs to treatment groups. There was no blinding of the treatment for the veterinarian that performed the evaluation of the outcome and this is a limitation of the study. Blinding was not considered practically achievable because of the different formulation of the two drugs. The fact that a binominal scale was chosen to measure treatment effect (number of affected claws before and after treatment) instead of a graduated scale or subjective measurements of improvement should decrease the risk for measurement error introduced by the lack of blinding. The hypothesis was that cyclosporine together with the diet would give a better treatment outcome compared to fish oil with diet, but after thirteen dogs, no significant difference between the two groups was observed. The post-hoc power calculation showed that the power to detect a difference in improvement in the magnitude of four claws between the two treatment groups was 30%, and that a doubling of the sample-size could have resulted in a power of 80% for this difference and significance level. However, because a difference of this size was considered unlikely to represent a biologically relevant effect the inclusion of additional patients was stopped.

## Conclusions

Cyclosporine and fish oil appeared to be equally effective in treating symmetrical onychomadesis when the dog is fed a diet high in omega-3. No statistically significant differences in the change in number of healthy claws were observed between dogs in the two treatment groups after six months of treatment. Symmetrical onychomadesis has been described in several other breeds and it is likely that the treatment regimens used in this trial could be used for dogs from other breeds as well. With long term follow-up, eleven of thirteen dogs continued to have onychomadesis or onychodystrophy after discontinuation of treatment and diet. However, the dogs appeared to live well with their disease after discontinuing treatment, although three dogs were not used for hunting because of discomfort. A diet rich in omega-3 may play an important role in suppressing the inflammatory reaction that takes place in the claw fold. Because of the high costs and lack of effect of cyclosporine compared to fish oil, we are inclined to suggest that omega-3 might be the first choice of treatment for canine symmetrical onychomadesis incorporated in the diet or as a supplement. However, the current study included a limited number of dogs and further investigation is warranted before treatment guidelines for the disease can be proposed.

## Abbreviations

BID: Bis in die- twice a day
DHA: Docosahexaenoic acid
DPA: Docosapentaenoic acid
EPA: Eicosapentaenoic acid
EVD: Eukanuba veterinary diets
FT4: Free thyroxin
TgAA: Thyroglobulin autoantibodies
TSH: Thyroid stimulating hormone
TT4: Total thyroxin
SLO: Symmetrical lupoid onychodystrophy
